# Exceptional response to alectinib for duodenal carcinoma with *ALK* fusion: A case report and literature review

**DOI:** 10.3389/fonc.2022.1064944

**Published:** 2023-01-12

**Authors:** Yuri Isaka, Akinori Sasaki, Akira Saito, Yasuaki Motomura, Yayoi Ando, Yoshiaki Nakamura

**Affiliations:** ^1^ Department of Gastroenterology, Tokyo Bay Urayasu Ichikawa Medical Center, Urayasu, Japan; ^2^ Department of Pathology, Tokyo Bay Urayasu Ichikawa Medical Center, Urayasu, Japan; ^3^ Clinical Research Support Office, National Cancer Center Hospital Chuou, Tokyo, Japan; ^4^ Department of Gastroenterology and Gastrointestinal Oncology, National Cancer Center Hospital East, Kashiwa, Japan

**Keywords:** duodenal cancer, *EML4-ALK* rearrangement, alectinib, next-generation sequencing, gastrointestinal cancer

## Abstract

Patients with advanced duodenal carcinoma usually have a poor prognosis due to limited effective chemotherapy options. The study for genotype-directed therapy in patients with duodenal carcinoma is progressing. However, no clinical data assessing the efficacy of molecularly targeted therapy are presently available. We report the case of a 64-year-old woman who was diagnosed with anaplastic lymphocyte kinase (*ALK*) fusion-positive advanced duodenal carcinoma. Echinoderm microtubule associated protein like-4 (*EML4*)*-ALK* rearrangement was detected by comprehensive genomic profiling after resistance to first-line chemotherapy. The patient received alectinib, an ALK inhibitor, with marked shrinkage in primary tumor and liver metastases. She is currently being treated with alectinib for 6 months or more. This is the first report of the efficacy of alectinib in a patient with duodenal carcinoma harboring *ALK* fusion. Additionally, this case report suggests that the practical use of next-generation sequencing may expand optimal treatment choices in rare solid tumors, including duodenal carcinoma.

## 1 Introduction

Small intestine cancer is a rare disease with a global incidence of less than 1 per 100,000 people (1). Duodenal carcinoma is the most common subtype of small intestine cancer, constituting more than 50% of small intestine cancers; it has the worst prognosis among small intestine cancers, with a one-year overall survival rate of approximately 20% (2). Although oxaliplatin or irinotecan-based systemic therapy is recommended for the treatment of the cancer in its advanced stages, there is limited evidence supporting the use of systemic therapy (3).

Multigene panel-based comprehensive genomic profiling (CGP) is widely used for the treatment of patients with malignant solid tumors. CGP enables the identification of patients with targetable alterations who may benefit from targeted therapies. A CGP study of small intestine cancer showed potentially targetable genomic alterations in the majority (91%) of cases, such as alterations in *KRAS*, *BRAF*, and *ERBB2* (4). Nevertheless, no targeted therapies have been established for the treatment of advanced small intestine cancer, excluding tumor-agnostic indications, such as pembrolizumab for microsatellite instability-high or tumor mutational burden-high tumors and TRK inhibitors for *NTRK* fusion-positive tumors. Therefore, presenting the efficacy of target therapies for patient-specific alterations is necessary for the development of effective targeted therapies for small intestine cancer, even if only one case has been reported.

Herein, we report a patient with anaplastic lymphoma kinase (*ALK)* fusion-positive duodenal carcinoma who achieved an exceptional response to alectinib. The patient provided informed consent for the presentation of anonymized clinical information.

## 2 Case description

**Figure 1:**
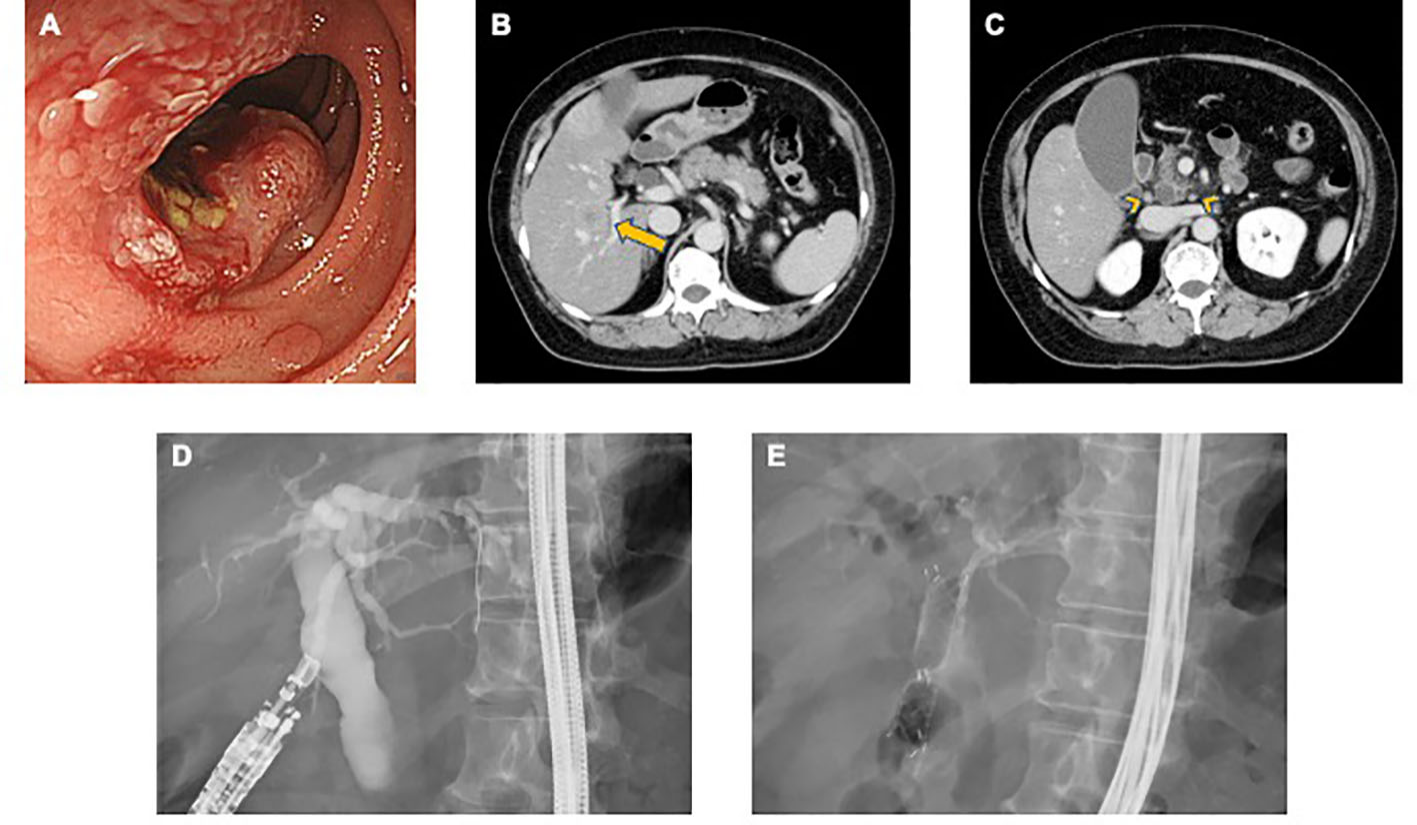
**(A)** Endoscopic findings of the duodenum reveal a type 2 tumor on the descending part. **(B, C)** Abdominal computed tomography shows multiple liver metastases (long arrow) and lymph nodes (arrowhead). **(D)** After EUS-guided puncture, contrast agent was injected into the common bile duct for cholangiogram. **(E)** Choledochoduodenostomy was accomplished with a fully covered metallic stent through the duodenal bulb.

A 62-year-old female patient presenting with epigastric pain and weight loss for several weeks was referred to the Tokyo Bay Urayasu Ichikawa Medical Center. The patient had a medical history of diabetes mellitus and bronchial asthma. Upper gastrointestinal endoscopy revealed Borrmann type 2 duodenal carcinoma in the descending part of the duodenum (**Figure 1A**). A biopsy specimen of the primary tumor revealed poorly differentiated adenocarcinoma. Computed tomography (CT) revealed multiple lymph nodes, liver metastases, and lung metastases (**Figures 1B, C**). The patient was diagnosed with stage IV duodenal carcinoma (clinical stage T3N2M1, according to the TNM classification, 8th edition). She received first-line chemotherapy with modified FOLFOX6 (mFOLFOX6) regimen every 2 weeks: oxaliplatin 85 mg/m^2^, leucovorin 400 mg/m^2^, 5-FU bolus 400 mg/m^2^, and continuous infusion of 5-FU 2400 mg/m^2^ over 46 h.

However, after four cycles of mFOLFOX6, the patient was admitted to the hospital for fever and abdominal pain. Laboratory findings showed elevated C-reactive protein (CRP) (4.34 mg/dL) and liver enzyme levels (aspartate aminotransferase [AST], 284 IU/L; alanine aminotransferase [ALT], 133 IU/L; total bilirubin, 5.67 mg/dL). Abdominal CT revealed biliary tract obstruction due to the progression of duodenal carcinoma and enlargement of liver metastases. The patient was diagnosed with acute cholangitis and administered antibiotics (4/0.5g of piperacillin-tazobactam 4 times daily). Thereafter, she underwent endoscopic ultrasonography-guided choledochoduodenostomy (EUS-CDS) for biliary drainage. Guide wire was inserted through the duodenal bulb into the common bile duct and a metallic biliary stent (fully covered metallic stent 6cm) was placed using EUS-CDS (**Figures 1D, E**). The patient’s fever went down, and her general condition gradually improved after the drainage procedure. The laboratory data, such as CRP and liver enzyme levels, were normalized (CRP, 0.24 IU/L; AST, 31 IU/L; ALT, 15 IU/L; total bilirubin, 0.84 mg/dL).

**Figure 2:**
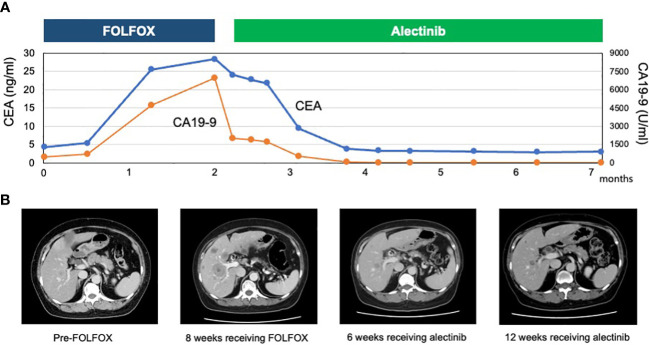
**(A)** Hematoxylin and eosin-stained biopsy specimen of the primary tumor. **(B)** Immunohistochemistry analysis showing strong ALK-positive staining.

The patient was considered to be refractory to mFOLFOX6 treatment. Subsequently, multigene panel-based CGP using FoundationOne CDx (Foundation Medicine, Cambridge, MA) was performed on the tumor tissue from the primary duodenal carcinoma. *ALK* fusion and echinoderm microtubule-associated protein-like 4 (*EML4*)-*ALK* rearrangement intron 19 were detected as actionable genomic alterations. Alterations in *CDKN2A/B* and *TP53* P151R were also detected; however, both were of unknown clinical significance. Immunohistochemistry analysis showed strong ALK expression in over 80% of tumor cells (**Figure 2**).

**Figure 3:**
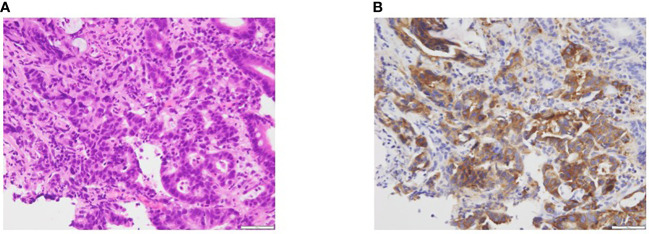
**(A)** Course of tumor markers (carcinoembryonic antigen [CEA] and carbohydrate antigen 19-9 [CA 19-9]) while receiving treatment with FOLFOX and alectinib. **(B)** Abdominal computed tomography showing multiple liver metastases pre-FOLFOX therapy, increased tumor volume 4 weeks after the FOLFOX therapy, and reduction of liver metastases 6 weeks after alectinib. the second follow-up CT imaging showed continuing response for liver metastases.

Based on the result of multigene panel-based CGP, the therapeutic strategy was discussed by an intra-institutional molecular tumor board, called an expert panel, and treatment with an ALK inhibitor was recommended. the patient started 300 mg of alectinib twice daily by participating in the BELIEVE trial (NCCH1901, jRCTs031190104) according to the expert panel’s recommendation. Several days after treatment initiation, there was a gradual improvement in the patient’s general condition with an associated reduction in the severity of abdominal pain. In addition, her performance status (PS) score improved from 1 to 0. The patient had grade 1 constipation and dysgeusia due to alectinib but tolerated the dose. A CT scan on day 49 showed a significant tumor reduction of liver lesions and lymph nodes, exhibiting a partial response (57% reduction), according to the Response Evaluation Criteria in Solid Tumors version 1.1 (**Figure 3**). Furthermore, the patient’s serum carcinoembryonic antigen and carbohydrate antigen 19-9 levels markedly decreased. The 2-month follow-up CT imaging, the second assessment of response to alectinib, showed continuing partial response. The patient returned to her weight before she got duodenal carcinoma. As of December 2022, the patient was still being treated with alectinib without tumor progression.

## 3 Discussion

We present the case of a patient with *ALK* fusion-positive duodenal carcinoma treated with alectinib. Our patient responded remarkably to alectinib treatment. *ALK* is located on chromosome 2p23 and encodes a transmembrane tyrosine kinase receptor. *ALK* rearrangements occur in the form of a translocation with another partner gene, leading to ALK activation. This fusion activates downstream signaling pathways, such as PI3K–Akt, MEKK2/3/MEK5/ERK5, RAS-MAPK, CRKL-C3G-RAP1, JAK-STAT, and JUN (5). Most *ALK* alterations account for *EML4-ALK* fusion. In addition, other *ALK* fusion partners include *STRN*, *KCNQ*, *KLC1*, *KIF5B*, *PPM1B*, and *TGF* (6). ALK inhibitors, including alectinib, have shown a significant survival benefit and have been approved for patients with non-small cell lung cancer (NSCLC) harboring *ALK* fusions. Alectinib is one of the most active ALK inhibitor showing a significant improvement of progression-free survival compared to crizotinib (7).

*ALK* translocations have been detected in other cancer types such as glioblastoma, non-Hodgkin lymphoma, and breast cancer (8, 9). However, the role of *EML4-ALK* as a therapeutic target in these non-NSCLC cancers, including gastrointestinal cancers, is not well understood. From our extensive literature search, we summarize in **Table 1** the case series of patients with gastrointestinal cancer who were treated with ALK inhibitors (10–17). A total of 23 patients with gastrointestinal cancer and *ALK* fusion were treated with ALK inhibitors. The median age was 50 years (range 32–87 years), and 13 patients (57%) were male. The cancer types included pancreatic (n = 10), colorectal (n = 8), gallbladder (n = 2), esophageal (n = 1), and gastric (n = 1), and only one patient with duodenal carcinoma was included. Crizotinib and alectinib were administered to 10 and 9 patients, respectively. The objective response rate to ALK inhibitors was 43.5%, and the median PFS was 6 months in patients in this literature review. The efficacy in these patients tended to be inferior to that in clinical trials involving patients with NSCLC harboring *ALK* fusions (7). One patient switched from crizotinib to ceritinib due to neutropenia, and no other serious adverse events were reported. Similar to our patient, the patient with duodenal carcinoma was treated with alectinib. Although the best response was stable disease, the patient achieved a PFS of 7.8 months.

**Table 1:** Review of ALK inhibitors for patients with gastrointestinal cancers.

Reference	Age	Sex	Primary tumor site	ALK fusion partner/breakpoint	First-line ALKi agent	TKI response	First-line ALKi PFS (month)
Gower A, 2020	41	F	Pancreas cancer	PPFIBP1	Alectinib	SD	5.0
Singhi AD, 2017	35	M	Pancreas cancer	EML4	Ceritinib	PR	7.0
Singhi AD, 2017	32	F	Pancreas cancer	EML4	Crizotinib	SD	17.0
Singhi AD, 2017	34	M	Pancreas cancer	STRN	Crizotinib	SD	10.0
Singhi AD, 2017	46	M	Pancreas cancer	EML4	Crizotinib	PD	2.0
Margherita A, 2022	75	M	Pancreas cancer	Intron 19 rearrangement	Alectinib	PD	0.9
Margherita A, 2022	34	M	Pancreas cancer	STRN	Crizotinib	PR	28.4
Margherita A, 2022	41	F	Pancreas cancer	PPFIBP1	Alectinib	PR	5.0
Margherita A, 2022	63	M	Pancreas cancer	EML4	Alectinib	SD	5.4
Margherita A, 2022	72	M	Pancreas cancer	EML4	Alectinib	NA	1.6
Hsiao SY, 2021	56	M	Colorectal cancer	EML4	Alectinib	PR	8.0
Amatu A, 2015	53	F	Colorectal cancer	CAD	Entrectinib	PR	4.5
Yakirevich E, 2016	87	F	Colorectal cancer	STRN	Ceritinib	SD	9.0
Margherita A, 2022	46	M	Colorectal cancer	EML4	Crizotinib	PD	2.3
Margherita A, 2022	45	M	Colorectal cancer	EML4	Crizotinib	PR	9.1
Margherita A, 2022	51	F	Colorectal cancer	CAD	Crizotinib	SD	3.7
Margherita A, 2022	50	F	Colorectal cancer	CAD	Entrecctinib	PR	4.6
Margherita A, 2022	67	M	Colorectal cancer	STRN	Alectinib	SD	13.6
Zhou Y, 2020	58	F	Gallbladder cancer	AMBRA1	Crizotinib	PR	7.0
Margherita A, 2022	58	F	Bile duct cancer	Unknown	Crizotinib	SD	3.9
Zhang X, 2021	36	M	Esophageal cancer	STRN	Crizotinib	PR	22.0
Margherita A, 2022	50	F	Gastric cancer	HMBOX1	Alectinib	PR	6.0
Margherita A, 2022	70	M	Duodenum cancer	KIF5B	Alectinib	SD	7.8

ALKi, anaplastic lymphoma kinase inhibitors; NA, not applicable; PR, partial response; SD, stable disease; PD, progressive disease; PFS, progression-free survival.

CGP studies for rare tumors identified targetable alterations, suggesting that the addition of CGP to management adds a potential line of therapy for tumors that have little or no standard of care (18). In addition, emergence of tumor-agnostic biomarkers, such as microsatellite instability-high, tumor mutational burden-high, and *NTRK1*/*2*/*3* fusions, supports the use of NGS for rare tumors. Since there is no randomized trial of systemic chemotherapy in patients with duodenal carcinoma due to its rarity, the choice of chemotherapy in duodenal carcinoma is also currently limited in clinical practice. With the development of next-generation sequencing (NGS), several meaningful oncogenic pathways have been found to be altered in duodenal carcinoma. In a CGP study, alterations in *ERBB2/HER2* and *BRAF*, microsatellite instability, and high tumor mutational burden were more frequently enriched in 317 small bowel cancers than that in gastric and colorectal cancers, and *EML4-ALK* fusion was identified in only one patient (0.3%) (4). Our case report encourages the use of NGS-based CGP for patients with small bowel cancers, including duodenal carcinoma, which potentially provides an opportunity of an appropriate precision medicine for these patients.

In conclusion, to our knowledge, this is the first report of an exceptional response to alectinib treatment in a patient with duodenal carcinoma harboring *ALK* fusion. The use of NGS to explore potentially actionable mutations in our patient paved the way for administering alectinib, which improved our patient’s quality of life and prolonged her survival after traditional chemotherapy failure. Therefore, NGS should be considered as a treatment option for patients with duodenal carcinoma. Further studies are warranted to evaluate the efficacy and safety of ALK inhibitors in patients with cancers other than NSCLC.

## Data availability statement

The original contributions presented in the study are included in the article/supplementary material. Further inquiries can be directed to the corresponding author.

## Ethics statement

Written informed consent was obtained from the individual(s) for the publication of any potentially identifiable images or data included in this article.

## Author contributions

YI, AkinS, and YN designed the study, collected data, performed data analysis, and wrote the manuscript. YM and YA were involved in data interpretation and critically reviewing the manuscript. AkirS was involved in testing tumor tissue as well as critically reviewing the manuscript. All authors read and approved the final manuscript.
